# Polychlorinated biphenyl exposure, diabetes and endogenous hormones: a cross-sectional study in men previously employed at a capacitor manufacturing plant

**DOI:** 10.1186/1476-069X-11-57

**Published:** 2012-08-29

**Authors:** Victoria Persky, Julie Piorkowski, Mary Turyk, Sally Freels, Robert Chatterton, John Dimos, H Leon Bradlow, Lin Kaatz Chary, Virlyn Burse, Terry Unterman, Daniel W Sepkovic, Kenneth McCann

**Affiliations:** 1 University of Illinois, School of Public Health, 1603 West Taylor St, Chicago, IL, 60612, USA; 2 Departments of Obstetrics, Gynecology and Physiology, Feinberg School of Medicine, Northwestern University, 710 N. Fairbanks Court, Olson Pavilion 8-408, Chicago, IL, 60611, USA; 3 Department of Research, Hackensack University Medical Center, 30 Prospect Avenue, Hackensack, NJ, 07601, USA; 4 Battelle Memorial Institute, 2971 Clairmont Road NE, Suite 450, Atlanta, Georgia, 30329, USA; 5 Department of Medicine, Section of Endocrinology, Diabetes, and Metabolism, University of Illinois, College of Medicine at Chicago, 820 South Damen Avenue, Chicago, IL, 60612, USA; 6 Jesse Brown VA Medical Center, 820 South Damen Avenue, Chicago, IL, 60612, USA; 7 Illinois Department of Public Health, Division of Environmental Health, 525 West Jefferson St, Springfield, IL, 62761, USA

**Keywords:** PCB, Hormones, Diabetes, Employees, Capacitor, Plant

## Abstract

**Background:**

Studies have shown associations of diabetes and endogenous hormones with exposure to a wide variety of organochlorines. We have previously reported positive associations of polychlorinated biphenyls (PCBs) and inverse associations of selected steroid hormones with diabetes in postmenopausal women previously employed in a capacitor manufacturing plant.

**Methods:**

This paper examines associations of PCBs with diabetes and endogenous hormones in 63 men previously employed at the same plant who in 1996 underwent surveys of their exposure and medical history and collection of bloods and urine for measurements of PCBs, lipids, liver function, hematologic markers and endogenous hormones.

**Results:**

PCB exposure was positively associated with diabetes and age and inversely associated with thyroid stimulating hormone and triiodothyronine-uptake. History of diabetes was significantly related to total PCBs and all PCB functional groupings, but not to quarters worked and job score, after control for potential confounders. None of the exposures were related to insulin resistance (HOMA-IR) in non-diabetic men.

**Conclusions:**

Associations of PCBs with specific endogenous hormones differ in some respects from previous findings in postmenopausal women employed at the capacitor plant. Results from this study, however, do confirm previous reports relating PCB exposure to diabetes and suggest that these associations are not mediated by measured endogenous hormones.

## Background

Polychlorinated biphenyls (PCBs) are a form of organochlorines that are very stable and resistant to temperature and pressure extremes. They were first commercially produced in the United States in 1929 and used widely in capacitors, transformers, hydraulic fluids, heat transfer fluids, lubricants, plasticizers and as components of surface coatings and ink until the USEPA banned its use in 1979. Employees at plants that used PCBs, such as capacitor manufacturing plants, were highly exposed with elevated levels that persist years after their employment [1]. In addition, PCBs are still present in many capacitors, transformers and other equipment manufactured before 1979, thus serving as a continuing source of indirect exposure for the general population, primarily through ingestion of contaminated food [2].

This paper focuses on associations of exposure to PCBs through previous employment at a capacitor manufacturing plant with diabetes and endogenous hormones. Diabetes has been associated with exposure to a wide variety of organochlorines, including dioxins and dioxin like compounds [3-10], PCBs [11-23], and persistent pesticides [20,22,24-30]. Mechanisms are not clear, with some [4,6,8], but not all [24,25,27,30,31], of these compounds thought to act through a specific aryl hydrocarbon (Ah) receptor. In addition, there is some evidence that effects may be stronger at lower levels of exposure [32,33] and may vary by obesity [27], as well as by other concurrent exposures.

PCBs also appear to act on a number of endogenous hormones, which in turn could mediate or confound relationships with diabetes. A recent paper of ours examined relationships of PCB exposure at a capacitor manufacturing plant with diabetes and endogenous hormones in post -menopausal women [18]. We found inverse associations of PCB levels with levels of sex-hormone binding globulin (SHBG), follicle stimulating hormones (FSH) and dehyrdroepiandrosterone sulfate (DHEAS). PCB exposure was also positively related to diabetes independent of potential confounders and measured hormones and diabetes was significantly and inversely associated with levels of triodothyronine (T3) uptake, FSH and DHEAS independent of PCB exposure [18]. This paper explores associations of PCB exposure with diabetes and endogenous hormones in men previously exposed at the same capacitor manufacturing plant.

## Methods

### Study design

This study is a cross-sectional examination of a sample of workers previously employed at the LaSalle Electrical Utilities Company (EUC), a capacitor manufacturing plant in Illinois [34] and a sample of local residents not previously employed at the plant. The study included a survey of previous exposures, life style factors and medical history as well as blood and urine measurements of PCBs, hormones, lipids, immune function and liver enzymes for 217 persons (68 men and 149 women, of whom 191 were former employees and 26 local residents not previously employed at the plant). The study was approved by the Human Subjects Institutional Review Board at University of Illinois at Chicago prior to enrollment.

### Participant selection

A total of 1,030 former employees were identified from union member lists from the 1980s, persons who attended community meetings in the 1980s and 1990s, and 400 payroll cards available to the investigators. A total of 251 persons were identified, located and approached for the study, of whom 191 agreed to participate. Unexposed persons were identified through random digit dialing of persons residing in the area, and included those who had lived in the area for more than 15 years, were aged *≥* 35 years and had never worked in the plant. A total of 26 of 146 eligible unexposed agreed to participate. This analysis is of the 63 men examined (after excluding two men on steroid medication and three men on thyroid medication).

### Exposure assessment

Exposure was classified into 8 main groups including total PCB concentration in serum, lipid-adjusted PCBs, total number of quarters worked at the plant determined from social security records, EUC job score (time of employment in each job weighted by exposure in each job category coded 1–4 depending on distance from the more highly exposed “cook department” (determined from a map of the plant and reported dates of work in specific jobs), dioxin-like PCBs (sum of congeners 105, 118, 156, 157, 167, 189), non dioxin-like PCBs (sum of all other PCB congeners analyzed (28, 52, 56/60, 66, 74, 99, 101, 110, 130, 137, 138, 146, 149, 153, 170, 171, 172, 177, 178, 180, 183, 187, 191, 193, 194, 195, 201, 203, 205, 206, 208 and 209) [35], estrogenic PCBs (sum of congeners 52, 99, 101, 110 and 153), and anti-estrogenic PCBs (the sum the congeners 105 and 156) [36]. Spearman correlations of PCB level with both quarters worked and job score were 0.77 and 0.76, respectively.

Individual congeners were analyzed as continuous variables if fewer than 16% of values were non-detectable (74, 99, 118, 138, 146, 153, 156, 170, 180, 187, 194, 201, 203, and 206).

### Surveys and collection of specimen

Participants were interviewed and fasting bloods taken in two phases during the summer of 1996. The survey was divided into two separate sessions administered by trained surveyers with the medical and lifestyle questions administered by a separate person than questions related to exposure so that each person was blinded as to the results of the other. Heights, weights and current medication use were determined at the time of fasting blood and urine collection and samples transferred to appropriate laboratories as described in the Additional file 1.

### Analyses of PCBs by CDC

PCBs were analyzed by capillary gas chromatographic method and compared on a subsample with column gas chromatographic method as described in Additional file 1. All total PCB levels presented in this report are the sum of the congeners: PCB #s 028, 052, 056/ 060, 066, 074, 099, 101, 105, 110, 118, 130, 137, 138, 146, 149, 153, 156, 157, 167, 170, 171, 172, 177, 178, 180, 183, 187, 189, 191, 193, 194, 195, 201, 203, 205, 206, 208, 209. Limits of detection for individual congeners are presented in the Additional file 1 (Additional file 1: Table S1). Values of PCBs below the limits of detection were imputed as zero.

### Analyses of chemistries, lipids, C-reactive protein and thyroid hormones by Smithkline Beecham

Serum glucose, triglycerides, cholesterol, gamma-glutamyl transferase (GGT), C-reactive protein (CRP), and thyroid hormones (total thyroxine (T4), free thyroxine index (FTI), triiodothyronine (T3), and thyroid stimulating hormone (TSH)) were analyzed by Smithkline-Beecham by methods described in Additional file 1. In analyses in this paper CRP was defined as moderate to high if levels were *≥* 1 mg/dL (10% in the sample of 63 men).

### Analyses of steroid hormones by Dr. Chatterton’s laboratory

Dehydroepiandrosterone sulfate (DHEAS), cortisol, luteinizing hormone (LH), and insulin were measured with radioimmunoassays; sex-hormone binding globulin (SHBG) and estradiol were measured using the Delphia system; testosterone was measured in serum by a coated tube assay obtained from Diagnostic Systems Laboratories, Webster, Texas; and SHBG-bound testosterone was determined as described by Bonfrer et al. [37] with details of procedures described in Additional file 1.

### Urine Analysis for 2-OHE_1_ and 16α-OHE_1_ by Dr. Bradlow’s Laboratory

Estrone metabolites, 2-hydroxyestrone (2-OHE1) and 16α – hydroxyestrone (16α-OHE1) were measured directly and concurrently in urine using enzyme immunoassay as described in Additional file 1.

### Insulin resistance and β-cell function

Serum insulin was not measured for any of the men with diabetes. Associations with insulin were therefore determined only for the 55 men without diagnosed disease for whom insulin levels were available and associations with the homeostatic model assessments of insulin resistance (HOMA-IR) and β-cell function (HOMA-%B) were calculated for the 52 men without diagnosed disease for whom glucose and insulin levels were available. HOMA-IR was defined as fasting glucose (mmol/L) x fasting insulin (mU/L)/22.5 after Wallace et al. [38]. HOMA-%B was estimated from (20xfasting insulin in mU/l)/(fasting glucose in mmol/L-3.5) after Wallace et al. [38].

### Diabetes

Diabetes was defined in these analyses as an affirmatory answer to the question “have you ever been diagnosed by a doctor as having diabetes mellitus or high blood sugar?” Eleven percent of men reported having diabetes (7 out of 63). Almost 60% of those with self-reported diagnosed diabetes were taking diabetes medication. Additional analyses were performed with different diabetes definitions, including those with diagnosed disease and men without diagnosed disease with fasting glucose levels *≥*126 mg/dL (n = 2 additional men) or fasting glucose *≥* 110 mg/dL (n = 8 additional men).

### Confounders

History of smoking and alcohol use was determined from the questions: “Are you currently smoking cigarettes?”, “On how many days of the past 30 days would you say you drank beer?..wine?..Liquor” and “On a typical day when you drank how much did you drink?” coded as an ordinal variable 1 = 0, 2 = 1-8, 3 > 8 drinks/month. Other potential confounders included age, body mass index (BMI) grouped into healthy weight, overweight and obese (1 = < 25 BMI in kg/m^2^, 2 = 25–29.9, and 3 = 30 or more), medication use, self-reported diagnosis of high blood pressure, CRP and GGT.

### Statistical analysis

Analyses for this report were restricted to men. Diabetes outcomes were defined as self-reported diagnosed diabetes, diagnosed diabetes and/or fasting glucose *≥* 126 mg/dL, and diagnosed diabetes and/or fasting glucose *≥* 110 mg/dL , as well as HOMA-IR, and HOMA-%B in men without diagnosed disease. Total serum lipids were calculated by the formula: total cholesterol (mg dL^-1^) X2.27 + triglycerides (mg dL^-1^) +62.3. Tests of normality using the Shapiro-Wilk statistic revealed that all exposure variables, and many endogenous hormones were not normally distributed. In these cases, the natural log transformed variable was used to achieve a more symmetric distribution, and geometric means used as the measure for descriptive purposes. For PCB exposure variables with a zero value, half of the lowest value above zero was added to all values prior to log transformation.

The association of exposure, potential confounders, and endogenous hormones with diagnosed diabetes was initially analyzed without adjustment for confounders of each factor separately. Small numbers precluded analyses within BMI strata. T-tests were performed for diabetes and continuous variables with geometric means and p-values presented. Chi-square odds ratios and p-values were assessed for dichotomous confounders (not presented). Logistic regression models were used when controlling for age, BMI group, and total lipids, with odds ratios and 95% confidence intervals presented.

Pearson correlations were used to analyze the relationship between continuous and ordinal potential confounders (including age, BMI group, GGT, lipids, number of drinks per month, and endogenous hormones), and exposure groups with and without controlling for age, BMI group and total lipids (partial correlation). T-test means and p-values also assessed the relationship between dichotomous potential confounders (current smoking, high blood pressure, and moderate to high CRP level) and exposure (not presented).

Logistic regression was used to generate adjusted odds ratios and p-values for diabetes with exposure, age, BMI group, total lipids, and relevant hormones. Multiple linear regression models assessed relationships of exposure to insulin, HOMA-IR and HOMA-%B with control for the potential confounders listed above. All analyses were performed using commercially available software (SAS version 9.2; SAS Institute Inc, Cary, North Carolina).

## Results

Table 1 presents the mean values for total PCBs, PCB groups, individual congeners, and potential confounders for men with and without diabetes, along with odds ratios for the relationships with diabetes controlling for age, BMI group and total lipids. Overall, 7/63 (11%) men were diagnosed with the disease, a rate comparable to the 12.3% currently seen in U.S. persons 45–64, but higher than seen 16 years ago at the time of examination [39]. Total PCBs, as well as each of the groups, and congeners 74, 99,118, 138,146,153,156,170,180, and 187 were significantly and positively related to diabetes, and remained associated after control for age, BMI group, and total lipids. Diabetes was not associated with smoking, high blood pressure, and elevated CRP (not shown).

**Table 1:** Relationships of exposures and potential confounders with diabetes in men, n = 63

	**Means**^**a**^			**Odds Ratios**^**c**^**(95% CIs)**
**Exposures and potential confounders**	**With**	**Without**	**P-value**	**Controlling for age, BMI group and total lipids**
	**diabetes**^**c**^	**diabetes**		
Age in years	58.3	55.6	.563	-
BMI Group	2.3	2.2	.753	-
Lipids	7.3	6.8	.430	-
Drink Group	1.6	2.2	.086	0.4 (0.2, 1.3)
GGT in U/L^d^	29.2	29.2	.994	0.9 (0.1, 5.3)
PCBs in ng/g^de^	19.6	4.7	.003	3.0 (1.3, 7.0)
Lipid PCBs in ng/g^d^	2741.1	709.1	.004	3.0 (1.3, 7.2)
Quarters Worked^d^	33.2	9.9	.070	1.8 (0.9, 3.7)
Job Score^d^	231.1	70.9	.116	1.6 (0.8, 3.1)
Dioxin-Like PCBs in ng/g^de^	2.5	0.4	.002	2.7 (1.3, 5.8)
Non-Dioxin PCBs in ng/g^de^	17.0	4.3	.004	3.0 (1.3, 7.2)
Estrogenic PCBs in ng/g^de^	3.6	1.0	.006	3.0 (1.2, 7.5)
Anti-Estrogenic PCBs in ng/g^de^	0.8	0.1	.006	2.4 (1.2, 4.9)
PCB074^d^	4.9	0.8	.014	2.1 (1.1, 3.9)
PCB099^d^	1.0	0.3	.003	3.4 (1.3, 9.0)
PCB118^d^	1.4	0.2	.002	3.0 (1.4, 6.8)
PCB138^d^	2.5	0.6	.003	2.9 (1.2, 6.6)
PCB146^d^	0.4	0.1	.003	2.7 (1.2, 5.8)
PCB153^d^	2.8	0.8	.005	3.1 (1.2, 7.8)
PCB156^d^	0.6	0.1	.007	2.3 (1.1, 4.7)
PCB170^d^	0.7	0.2	.007	3.1 (1.2, 7.9)
PCB180^d^	1.1	0.4	.011	3.3 (1.2, 9.3)
PCB187^d^	0.3	0.1	.023	2.4 (1.05, 5.4)
PCB194^d^	0.2	0.1	.069	2.1 (0.9, 5.0)
PCB201^d^	0.2	0.1	.055	2.4 (0.9, 6.5)
PCB203^d^	0.2	0.1	.067	2.4 (0.9, 6.8)
PCB206^d^	0.1	0.04	.469	1.3 (0.5, 2.9)

^a^ Geometric mean presented for those variables natural log-transformed.

^b^Adjusted odds ratio controls for age, BMI group, and total lipids (except in the case of lipid-adjusted PCBs which does not control for lipids).

^c^A total of 7 men had diagnosed diabetes and 56 men were without a diagnosis of diabetes.

^d^Variable not normally distributed, therefore natural log-transformed variable used in logistic regression model.

^e^PCB is a sum of all PCB congeners measured, <LOD = zero; Dioxin-like PCBs include 105, 118, 156, 157, 167, 189; Non dioxin-like PCBs: sum of all other PCBs beside dioxin-like; Estrogenic PCBs: sum of 52, 99, 101, 110, 153; Anti-estrogenic PCBs: sum of 105, 156.

Table 2 presents relationships of endogenous hormones with diabetes. The only hormone that was significantly related to diabetes was 16α-OH estrone which was inversely associated after control for age, BMI group, and total lipids.

**Table 2:** **Relationships of endogenous hormones with diabetes in men, n = 63**^**a**^

	**Means**^**b**^			**Odds Ratios**^**c**^**(95% CIs)**
**Endogenous hormone**	**With**	**Without**	**P-value**	**Controlling for age, BMI group and total lipids**
	**diabetes**^**d**^	**diabetes**		
TSH ultra sens in μIU/mL^e^	1.4	1.7	.192	0.3 (0.1, 2.0)
Triiodothyronine in ng/dL^e^	121.8	124.3	.693	0.1 (0.0, 118.9)
T3-uptake in%	29.6	31.2	.261	0.9 (0.7, 1.1)
Total T4 in μg/dL^e^	7.9	7.3	.238	17.8 (0.1, >999.9)
Free T4 Index^e^	2.3	2.3	.760	1.7 (0.0, 319.9)
SHBG in nmol/L^e^	33.5	40.8	.427	0.5 (0.1, 2.5)
LH in mIU/ml^e^	4.2	4.8	.490	0.6 (0.1, 2.5)
Testosterone (T)^e^	17.8	19.6	.539	0.8 (0.1, 7.0)
% T bound to SHBG	29.6	30.1	.860	1.0 (0.8, 1.1)
Conc. T bound to SHBG^e^	5.1	5.7	.575	0.8 (0.1, 4.3)
Estradiol (E2) in pmol/L^e^	143.7	126.7	.267	7.0 (0.3, 153.7)
DHEA Sulfate in μmol/L^e^	7.1	5.4	.463	2.1 (0.6, 7.9)
Cortisol in nmol/L^e^	631.9	507.8	.191	5.1 (0.6, 46.9)
2-OHE1 in ng/mL^e^	4.7	7.2	.063	0.2 (0.0, 1.2)
16a-OHE1 in ng/mL^e^	2.9	5.0	.024	0.1 (0.0, 0.8)
2-OHE1/16a-OHE1^e^	1.5	1.5	.895	1.3 (0.2, 10.2)
2-OHE1/Creatinine^e^	5.6	5.2	.759	1.5 (0.3, 7.2)
16a-OHE1/Creatinine^e^	3.7	3.6	.959	1.2 (0.2, 7.0)

^a^ Hormones treated as continuous variables and analyzed with and without control for age, BMI group, and total lipids in logistic regression.

^b^Geometric mean presented for those variables natural log-transformed.

^c^Adjusted odds ratio controls for age, BMI group, and total lipids.

^d^A total of 7 men had diagnosed diabetes and 56 men were without a diagnosis of diabetes.

^e^Variable not normally distributed, therefore natural log-transformed variable used in logistic regression model.

Table 3 presents relationships of total PCBs, lipid adjusted PCBs, PCB groups and job scores with endogenous hormones and potential confounders. Most PCB groups (except for estrogenic PCBs) were significantly and inversely associated with TSH and several PCB and job exposure groups were inversely associated with T3-uptake, while job exposure groups, but not PCBs, were positively associated with total T4, percent testosterone bound to SHBG, and cortisol. Estrogenic PCB group was significantly and positively associated with DHEAS after control for age, BMI group and total lipids. None of the exposure groups were significantly associated with T3, free T4 index, SHBG, LH, testosterone, or concentration of percent of testosterone bound to SHBG. Nor were they significantly associated with estradiol, 2-OH estrone, or 16α-OH estrone. After control for age, BMI group and total lipids, congeners 74, 146, and 156 were negatively and significantly related to TSH, while relationships with 99, 138, 153 and 170 were of only borderline significance (p-value ≥ .05 but less than .10) (Additional file 1: Table S2). The congeners negatively and significantly related to T3-uptake include 74, 170, and 203. Inverse associations with 138, 156, 180, 187 and 201 were of borderline significance. None of the other individual congeners were related to T3-uptake. Congeners 138, 153 and 180 were positively associated with DHEAS. In addition, positive relationships were seen with several congeners and testosterone (153, 170, and 180) and concentration of T bound to SHBG (170 and 180). There were no other significant relationships of individual congeners with other hormones after control for age, BMI group and total lipids (Additional file 1: Table S2)..

**Table 3:** Relationships of potential confounders with EUC exposure variables, Pearson correlation coefficient, n = 63

	**PCBs**^**ab**^	**Lipid**	**Qrts**	**Job**	**Dioxin-like**	**Non-dioxin**	**Estro-genic**	**Anti-estrogenic**
		**PCBs**^**b**^	**worked**^**b**^	**score**^**b**^	**PCBs**^**ab**^	**PCBs**^**ab**^	**PCBs**^**ab**^	**PCBs**^**ab**^
Age in years	.31^c^	.31^c^	.21	.18	.39^c^	.30^c^	.33^c^	.42^c^
BMI Group	-.03	-.09	-.14	-.15	.00	-.04	.05	-.05
Lipids	.20	.03	.11	.01	.22	.20	.24	.19
Drink Group	-.19	-.18	-.09	-.10	-.20	-.19	−08	-.18
GGT in U/L^b^	.04	.01	-.01	.01	-.02	.05	-.02	-.30
TSH ultra sens in mciu/mL^b^	-.29^cd^	-.27^cd^	-.37^cd^	-.36^cd^	-.26^ce^	-.29^cd^	-.25^e^	-.30^cd^
Triiodothyronine in ng/dL^b^	-.03	-.10	.01	.02	-.08	-.03	-.01	-.06
T3-uptake in%	-.27^cd^	-.25^d^	-.24^d^	-.28^cd^	-.17	-.28^cd^	-.21^e^	-.17
Total T4 in mcg/dL^b^	.14	.11	.26^cd^	.28^cd^	.12	.14	.19	.11
Free T4 Index^b^	-.03	-.04	.15	.15	.01	-.04	.06	.01
SHBG in nmol/L^b^	-.00	.06	.06	.11	.02	-.00	-.06	.06
LH in mIU/ml^b^	-.13	-.11	.08	.11	-.15^e^	-.13	-.13	-.12
Testosterone (T) in nmol/L^b^	-.05	.01	-.08	-.02	-.01	-.06	.03^e^	-.01
% T bound to SHBG	.10	.16	.18	.25^c^	.09	.10	.08	.15
Conc. T bound to SHBG^b^	-.00	.08	.02	.10	.02	-.00	.06	.06
Estradiol (E2) in pmol/L	.13	.16	.01	-.01	.12	.13	.16^e^	.08
DHEAS in μmol/L^b^	-.02	-.03	-.04	-.03	.04	-.02	.13^d^	.00
Cortisol in nmol/L^b^	.16	.17	.27^cd^	.25^ce^	.17	.16	.14	.16
2-OHE1 in ng/mL^b^	.01	-.00	.06	.11	-.02	.01	.03	.03
16a-OHE1 in ng/mL^b^	-.01	-.04	-.04	-.03	.01	-.01	.02	-.01
2-OHE1/16a-OHE1^b^	.00	.02	.13	.17	-.06	.01	-.01	.02
2-OHE1/Creatinine^b^	.04	.07	.14	.21	.02	.05	-.03	.11
16a-OHE1/Creatinine^b^	.02	.05	.04	.09	.03	.02	-.06	.06

^a^PCB is a sum of all PCB congeners measured, <LOD = zero: 28, 52, 56/60, 66, 74, 99, 101, 105, 110, 118, 130, 137, 138, 146, 149, 153, 156, 157, 167, 170, 171, 172, 177, 178, 180, 183, 187, 189, 191, 193, 194, 195, 201, 203, 205, 206, 208, 209; Dioxin-like PCBs: 105, 118, 156, 157, 167, 189;Non dioxin-like PCBs: sum of all other PCBs beside dioxin-like; Estrogenic PCBs: sum of 52, 99, 101, 110, 153; Anti-estrogenic PCBs: sum of 105, 156.

^b^variable not normally distributed, therefore natural log-transformed variable used.

^c^p-value < .05 in unadjusted analysis.

^d^p-value < .05 after control for age, bmi group and total lipids (note that with lipid-adjusted PCBs, total lipids is not controlled for).

^e^p-value ≥ .05, but less than .10 after control for age, bmi group and total lipids

Table 4 presents final models of exposure, age, BMI group and total lipids against diabetes. Most exposure groups (aside from quarters worked and job score) remain significant predicators after control for potential confounders. The associations remained significant with only PCB exposure and total lipids, as well as with 16α-OH estrone, in the model (not shown). Addition of each of the thyroid hormones (TSH, T3, total T4, free T4 or T3-uptake) separately to models with exposure group, age, BMI group, and lipids did not change the exposure relationships with diabetes (not shown). Many of the individual congeners were positively and significantly associated with diabetes after control for age, BMI group, and lipids (74, 99, 118, 138, 146, 153, 156, 170, 180 and 187). There were not significant associations with the more highly chlorinated congeners (194, 201, 203, 206).

**Table 4:** **Multivariable models of diabetes in men: odds ratios and 95% confidence intervals presented for PCB exposure, age, BMI group, and lipids n = 63**^**a**^

**Variables in model**	**PCBs**^**bc**^	**Lipid**	**Qrts**	**Job**	**Dioxin-**	**Non-**	**Estrogenic**	**Anti-**
		**PCBs**^**b**^	**worked**^**b**^	**score**^**b**^	**like**	**dioxin**		**estrogenic**
					**PCBs**^**bc**^	**PCBs**^**bc**^	**PCBs**^**bc**^	**PCBs**^**bc**^
EUC exposure^d^	3.0	3.0	1.8	1.6	2.7	3.0	3.0	2.4
	(1.3, 7.0)	(1.3, 7.2)	(0.9, 3.7)	(0.8, 3.1)	(1.3, 5.8)	(1.3, 7.2)	(1.2, 7.5)	(1.2, 4.9)
Age	1.0	1.0	1.0	1.0	1.0	1.0	1.0	1.0
	(0.9, 1.1)	(0.9, 1.1)	(0.9, 1.1)	(0.9, 1.1)	(0.9, 1.1)	(0.9, 1.1)	(0.9, 1.1)	(0.9, 1.1)
BMI Group	1.5	1.7	1.4	1.4	1.4	1.5	1.2	1.6
	(0.3, 6.5)	(0.4, 7.2)	(0.4, 5.5)	(0.4, 5.1)	(0.3, 6.1)	(0.4, 6.6)	(0.3, 5.2)	(0.4, 6.5)
Lipids	1.1	n/a	1.2	1.3	1.0	1.1	1.1	1.1
	(0.5, 2.1)		(0.6, 2.2)	(0.7, 2.4)	(0.5, 2.1)	(0.5, 2.1)	(0.6, 2.1)	(0.5, 2.1)

^a^ Logistic Regression models included variables listed in 1^st^ column.

^b^variable not normally distributed, therefore natural log-transformed variable used in logistic regression model.

^c^PCB is a sum of all PCB congeners measured, <LOD = zero; Dioxin-like PCBs include: 105, 118, 156, 157, 167, 189; Non dioxin-like PCBs: sum of all other PCBs beside dioxin-like; Estrogenic PCBs: sum of 52, 99, 101, 110, 153; Anti-estrogenic PCBs: sum of 105, 156.

^d^EUC Exposure refers to the exposure variable listed in the column headings of the table.

Additional analyses were done including the 2 men without diagnosed disease with fasting glucose levels *≥*126 mg/dL and including the 8 men without diagnosed disease with fasting glucose levels *≥* 110 mg/dL (not shown). All PCB groups, except for antiestrogenic PCBs, were significantly higher in the 9 men with diabetes or glucose levels *≥* 126 mg/dL after control for age, BMI group, and lipids. Many of the lower chlorinated congeners (99, 118, 138, 146, 153, 170, 180) were significantly related to those men with diabetes or glucose ≥ 126 mg/dL after control for age, BMI group and lipids. None of the PCB groups or individual congeners were significantly different between 15 men with diabetes or fasting glucose *≥*110 mg/dL without a diagnosis and those without diabetes on unadjusted analyses or after control for potential confounders.

None of the exposure variables were related to insulin resistance as measured by HOMA-IR (Table 5) with or without control for potential confounders. Only BMI group was positively and significantly related to HOMA-IR. Similarly, none of the individual congeners were significantly related to HOMA-IR (not shown).

**Table 5:** **Multivariable models of HOMA-IR in men without diabetes: parameter estimates and 95% confidence intervals for PCB exposure group, age, BMI group and lipids, n = 52**^**a**^

**Variables in model**	**PCBs**^**bc**^	**Lipid**	**Qrts**	**Job**	**Dioxin-**	**Non-**	**Estrogenic**	**Anti-**
		**PCBs**^**b**^	**worked**^**b**^	**score**^**b**^	**like**	**dioxin**		**estrogenic**
					**PCBs**^**bc**^	**PCBs**^**bc**^	**PCBs**^**bc**^	**PCBs**^**bc**^
EUC exposure^d^	-.02	-.01	-.03	-.03	-.02	-.02	-.03	-.03
	(−.13, .10)	(−.13, .10)	(−.11, .04)	(−.09, .04)	(−.11, .07)	(−.14, .10)	(−.14, .09)	(−.11, .06)
Age	-.00	-.00	-.00	-.00	-.00	-.00	-.00	-.00
	(−.01, .01)	(−.01, .01)	(−.01, .01)	(−.01, .01)	(−.01, .01)	(−.01, .01)	(−.01, .01)	(−.01, .01)
BMI Group	.37	.39	.36	.36	.36	.37	.37	.36
	(.19, .55)	(.22, .56)	(.18, .54)	(.18, .54)	(.18, .55)	(.19, .55)	(.19, .55)	(.18, .54)
Lipids	.04	n/a	.04	.03	.04	.04	.04	.04
	(−.05, .13)		(−.05, .13)	(−.05, .12)	(−.05, .13)	(−.05, .13)	(−.05, .13)	(−.05, .13)

^a^Analyses excluded men without a diagnosis of diabetes who did not have insulin or glucose measurements. Multivariable regression models included variables listed in 1^st^ column.

^b^variable not normally distributed, therefore natural log-transformed variable used in regression model.

^c^PCB is a sum of all PCB congeners measured, <LOD = zero; Dioxin-like PCBs include: 105, 118, 156, 157, 167, 189; Non dioxin-like PCBs: sum of all other PCBs beside dioxin-like; Estrogenic PCBs: sum of 52, 99, 101, 110, 153; Anti-estrogenic PCBs: sum of 105, 156.

^d^EUC Exposure refers to the exposure variable listed in the column headings of the table.

As with HOMA-IR, BMI group was positively and significantly related to both HOMA-%B and insulin (not shown). None of the exposure variables (groups or individual congeners), however, were related to either HOMA-%B or insulin (analyzed separately) with or without control for potential confounders (not shown). The ratio of 2-OHE1 divided by creatinine was the only endogenous hormone measured that was significantly and positively related to HOMA-%B after control for age, BMI group, total lipids and exposure. None of the endogenous hormones were related to insulin after control for the same covariates (not shown).

## Discussion

Findings from this study are consistent with previous reports that have found positive associations of PCBs with diabetes [11-17,20-23]. The lack of association with insulin resistance is also consistent with our previous report in women exposed at the same capacitor manufacturing plant [18]. Associations with endogenous hormones, while not affecting the relationships of PCB with diabetes, differ in some respects from findings in postmenopausal women from the same study [18]. While the inverse association of PCB with T3 uptake was also seen in women from that study, the inverse association with TSH was not seen in our previous paper, nor were the positive associations of job score and quarters with total T4 [18].

Other studies have found associations of PCBs with thyroid hormones although the direction and magnitude of these associations have not been consistent, in part because of variations in populations, exposure levels, and types of exposure. Studies in adults and adolescents with low to moderate PCB exposures have usually [40-46], but not always [47,48], shown decreased levels of peripheral thyroid hormones without consistent changes in TSH and with effects generally stronger in women than in men [41,42,44,46]. The National Health and Nutrition Examination study found positive associations of PCBs with TSH in older women, but negative associations of PCBs with TSH in older men [46]. Gender differences were also seen in Canada where PCBs were inversely associated with T3 but not T4 in women, and PCBs were inversely associated with T4 but not T3 in men [40].

Thyroid associations with other forms of PCB exposure are not consistent, but tend to show positive, rather than negative, associations with peripheral thyroid hormones at higher levels of exposure [49-52]. Exposures from industrial sources, like the PCB and chlorinated naphthalene exposure in the current study, are often mixed with other exposures from dioxin like compounds and there is evidence that dioxin-like compounds have different effects than non-dioxin like PCBs that may have, in part, account for some of the differences seen among the studies, as noted in a cohort of occupationally exposed persons, whose exposure was primarily to dioxins, where workers had significantly higher levels of FT4 than the referent group [44]. Turyk et al., using data from Great Lakes male fish consumers, examined associations of PCBs and TEQs separately and together and found that, although both PCBs and TEQs were inversely associated with T4 when analyzed separately, the inverse associations were stronger in men with higher PCBs and lower TEQs [53].

The inverse association of T3 uptake with PCB level reported here for men was also seen with PCB level in our previous publication in women [18]. An earlier study, however, in consumers of Great Lakes fish noted a positive association of T3 uptake with PCB level in men and no association with PCB level in women, again suggesting varying effects from different mixtures of exposure [44]. T3 uptake is inversely related to thyroid binding globulin (TBG), which, in turn, is positively associated with estrogenization [54]. Changes in binding proteins affect proportions of free thyroid hormones which are active at the tissue level. Animal studies have shown strong inverse associations of PCBs with T4, in part related to decreased binding to tranthyretin [55], which accounts for binding of only 10% of T3 and T4 in adult humans. Other studies have not shown consistent associations with TBG [52,56,57].

No associations were seen in the current study of total PCBs with testosterone, LH, FSH, estradiol, DHEAS, SHBG or cortisol, although positive associations with DHEAS, testosterone, and the percent testosterone bound to SHBG were seen with selected congeners. Previous studies in men have not found consistent associations of PCBs with these hormones. No significant association was seen of PCBs with estradiol, total testosterone, or SHBG in male fishermen or in men age 41–55 environmentally exposed from Slovakia [41,47,58]. However, significant inverse associations with testosterone were seen in a larger group in Slovakia [58], weak inverse associations were seen for PCB153 with free testosterone with men undergoing pre-military service examination [59], inverse associations of total PCBs with testosterone were seen in adult Native American men [60], and inverse associations of borderline significance were seen for dioxin-like PCBs with total testosterone in men of reproductive age [61]. Inconsistent relationships were seen with gonadotropin, SHBG, estradiol and testosterone levels in Inuits and 3 European cohorts, where positive associations of PCB 153 were seen with SHBG and LH in some but not all groups [62]. Our previous study in Great lakes fish consumers noted significant inverse associations of PCBs with SHBG-bound testosterone, but no association of PCBs with estrone sulfate, FSH, LH, DHEAS, SHBG or free testosterone, suggesting that PCBs may affect the binding of testosterone, rather than the level [44]. Inverse associations were stronger in participants with higher PCB levels and lower TEQs [53].

The inverse relationship of 16α-OH estrone with diabetes could be secondary to more diluted urine in diabetics, a possibility supported by the lack of association after control for urine creatinine levels. 16α-OH estrone is not a hormone generally examined in relation to clinical diabetes and the association with diabetes was not seen in our previous paper from women in the same cohort [18]. There is some evidence that PCB exposure is positively associated with 16/2-OH estrone [63], although none of the measures of PCB exposure in the current analysis were related to 16α-OH estrone and the relationships with diabetes were independent of exposure. 16α-OH estrone is generally thought to be more estrogenic than 2-OH estrone [63] and endogenous estrogens have been positively associated with diabetes in men as well as women [64]. The current finding of an inverse association is therefore counterintuitive and more likely related to urinary dilution than a biologic effect.

Previous studies have suggested that endogenous androgens in men are inversely associated with diabetes [64,65]. The current study did not find associations of diabetes with testosterone or with the concentration bound to SHBG. However, we did not examine dihydrotestosterone or glucuronidated androgen metabolites, which were positively associated with diabetes in a recent study of older black men [65]. This study also did not find an inverse association of diabetes with DHEAS, nor did it see associations of PCB exposure with SHBG or DHEAS which were seen in the recent analyses from women from the same cohort [18], suggesting gender differences in the relationships of both PCB exposure and diabetes with endogenous steroid hormones.

The lack of association with insulin resistance as defined by HOMA IR in those without diagnosed disease, as well as the lack of association when persons with glucose ≥110 mg/dL are included in the exposed group, is consistent with our previous report from women in the same cohort [18] and suggest effects on β-cell function [66]. The few other studies that have examined relationships of PCBs with HOMA-IR have not reported consistent results. Positive associations were seen in NHANES in non-diabetics, with an inverted U shaped dose response [67,68]. Others have seen positive associations of HOMA-IR with pesticides but negative associations with PCBs [69]. Still others have found no significant associations with PCBs [70].

The small number of men with diabetes in this study is a limitation. In particular, it would have been of interest to explore whether relationships with diabetes were modified by level of obesity or by endogenous hormone levels. A second limitation is the cross-sectional nature of the study design. There is the possibility that existing diabetes could modify PCB levels through altered metabolism, although previous studies of PCBs and dioxins do not support that hypothesis [15,30,71]. Other limitations include the single time of biologic sample collection, lack of data on other exposures, such as chlorinated naphthalenes, imprecise measures of insulin resistance and secretory capacity, and limited data on additional confounders and effect modifiers.

## Conclusions

Overall, however, this study is consistent with an increasing body of literature supporting positive associations of organochlorine exposure with diabetes in men and women. It also suggests associations of occupational exposures with hormone regulation that vary by gender and specific exposure group and implies biologic pathways by which these hormones may be operative. The results have implications for the general population today. Although PCB levels are decreasing, exposures persist, in part through contaminated food. The findings underscore the importance of continued surveillance and dietary recommendations in protecting the public health of the overall population.

## Abbreviations

Ah, Aryl hydrocarbon; BMI, Body mass index; CRP, C-reactive protein; DHEAS, Dehydroepiandrosterone sulfate; EUC, LaSalle Electrical Utilities Company; FSH, Follicle stimulating hormone; FTI, Free thyroxine index; GGT, Gamma-glutamyl transferase; HOMA-IR, Homeostatic model assessment of insulin resistance; HOMA-%B, Homeostatic model assessment of beta cell function; LH, Luteininzing hormone; NHANES, National Health and Nutrition Examination Survey; 2-OHE1, 2-hydroxyestrone; 16-OHE1, 16α-hydroxyestrone; PCB, Polychlorinated biphenyl; SHBG, Sex hormone binding globulin; T3, Triiodothyronine; T4, Thyroxine; TBG, Thyroxine binding globulin; TSH, Thyroid stimulating hormone.

## Competing interests

The authors declare that they have no competing interests.

## Authors’ contributions

VP and KM, as Principal Investigators of the study, had overall responsibility for the study design, implementation and interpretation; LC worked with VP and KM on the original hypotheses and directed the field work; JP, MT and SF directed the data analysis; RC, HB, and DS were responsible for the hormone analyses; VB performed PCB exposure analyses; and JD was responsible for the EUC job exposure classifications. All authors have been involved in drafting the manuscript and given approval for its publication.

## Supplementary Material

Additional file 1 Additional details on laboratory methods. Click here for file
