# Supplementary material for: Polychlorinated biphenyl exposure, diabetes and endogenous hormones: a cross-sectional study in men previously employed at a capacitor manufacturing plant
Source: Environ Health. 2012 Aug 29;11:57. doi: 10.1186/1476-069X-11-57 (PMC3476446; doi:10.1186/1476-069X-11-57)
Supplement: Additional file 1 — Additional details on laboratory methods. [file 1476-069X-11-57-S1.doc]

**SUPPLEMENTAL MATERIAL**

**Polychlorinated Biphenyl Exposure, Diabetes and Endogenous Hormones: A Cross-Sectional Study in Men Previously Employed at a Capacitor Manufacturing Plant**

Victoria Persky, Julie Piorkowski, Mary Turyk, Sally Freels, Robert Chatterton Jr, John Dimos, H. Leon Bradlow, Lin Kaatz Chary, Virlyn Burse, Terry Unterman, Daniel Sepkovic, Kenneth McCann

**Collection of blood and urine specimens**

Fasting blood specimens were collected by trained technicians. One 5 mL EDTA lavender stopper tube and 10 mL of whole blood were refrigerated and sent daily to Smithkline Beecham for measurements of chemistries, thyroid hormones and CBC analysis; 10 mL of serum, one 5 mL EDTA lavender stopper tube and one 10 mL heparanized tube were sent to CDC for PCB and immune measurements; and 10 mL of serum was frozen and sent to Dr. Robert Chatterton’s laboratory at Northwestern University for measurements of steroid hormones. First morning urine specimens were collected at home the day of the examination in a cup containing 100 mg vitamin C; 15 cc were then frozen and sent to Dr. Bradlow’s laboratory for measurement of urine metabolites.

**Analyses of PCBs by CDC**

Two separate methods were used by CDC to analyze for PCBs. A packed column gas chromatographic method using technical Aroclors 1242 and/or 1260 as standards and reported as total PCBs (as Aroclor 1242 and/or Aroclor 1260) and total PCBs (the sum of Aroclor 1242 and Aroclor 1260). A capillary gas chromatographic method provided concentration levels for 38 individual PCB congeners, the sum of which was reported as total PCBs. We compared the two analytical approaches using a subset of samples (n=45) in an attempt to determine the comparability of the two methods and found a Spearman correlation coefficient of 0.925 (p<0.001). Subsequently, all remaining samples were analyzed for specific PCB congeners using the capillary gas chromatographic method described below. All total PCB levels presented in this report are the sum of the congeners: PCB #s 028, 052, 060, 066, 074, 099, 101, 105, 110, 118, 130, 137, 138, 146, 149, 153, 156, 157, 167, 170, 171, 172, 177, 178, 180, 183, 187, 189, 191, 193, 194, 195, 201, 203, 205, 206, 208, 209. Limits of detection for individual congeners are given in Table 1S. Values below the limits of detection were assumed to be zero.

All samples first underwent extraction and clean up. Serum was denatured with methanol and extracted with hexane:ethyl ether (1:1). The extracts were eluted through adsorption silica gel (deactivated to 3% with water) with hexane. Surrogates used to monitor the capillary analytical process were Ballschmiter and Zell (BZ) [1] #30 (2,4,6-trichlorobiphenyl), PCB congener BZ #204 (2,2',3,4,4',5,6,6'-octachlorobiphenyl) and hexabromobiphenyl (2,2',4,4',5,5'-hexabromobiphenyl). The recovery of surrogates was not used to correct analytical results reported for unknowns.

All specimens were analyzed by the capillary method using a 60mx0.25mm i.d. x 0.25um film thickness DB-5 and 60 m x 0.25 mm i.d. x 0.25 um film thickness DB-1701 (J&W Scientific, Folsom, CA). A Hewlett/Packard 5890 GC equipped with a HP7673 autosampler was used (Hewlett-Packard, Wilmington, DE). Individual congeners (Ultra Scientific, North Kingston, RI) were used as standards with response factors being generated relative to the internal standard (1,2-dichloronaphthalene; Ultra Scientific, North Kingston, RI). Data were handled by a P-E Nelson Turbochrom Chromatography Data Station (The Perkin-Elmer Corporation, San Jose, CA). Serum pools, made from bovine serum that contained in vivo PCBs as Aroclor 1242 or Aroclor 1260 derived from goats fed these technical materials, were used as quality control materials [2]. These pools were characterized through repetitive analysis (n=20) for individual congeners setting mean, 95% and 99%, control limits. Pools analyzed with unknowns had to meet quality control limits before data were reported.

**Measurements of cholesterol, triglycerides, GGT, C-reactive protein and thyroid hormones by Smithkline- Beecham.**

*Lipids and glucose* were determined on an Olympus AU 5200 by Smithkline Beecham.

*Cholesterol:* For this analysis, cholesterol esters present in the serum were hydrolyzed to free cholesterol and fatty acids by cholesterol esterase. The cholesterol was then oxidized by cholesterol oxidase to cholest-4-en-3-one with the simultaneous production of hydrogen peroxide, which in turn reacted with 4-aminoantipyrine to produce a chromophore. The resulting absorbance of the reaction mixture was measured bichomatically at 540/600 nm and was proportional to the cholesterol concentration in the serum.

*Triglcerides.* For this analysis triglyceride was hydrolyzed to free fatty acids and glycerol by lipase. The glycerol was then enzymatically phosphorylated and then oxidized with glycerol phosphate oxidase. The resultant hydrogen peroxide produced reacted with the chromogen 4-aminoantipyrine to give a chromophore which was measured bichromatically at 520/660 nm. The increase in absorbance of the reaction mixture was directly proportional to the triglyceride concentration in the serum.

*Gamma-glutamyl transferase (GGT)*: In this assay GGT catalyzed the transfer of an L-glutamyl group from the substrate L- gamma-glutamyl-3-carboxyl-4 nitroanilide to glycylglycine to form 5-amino-2-nitrobenzoate resulting in a change in absorbance 410/480 nm.

*C Reactive Protein (CRP)*: CRP was measured by laser turbidometry, calibrated to the U.S. National Reference Preparation maintained at the Centers for Disease Control and Prevention [3]. In analyses in this paper CRP was defined as moderate to high (10% of men) if levels were > 1 mg/dL.

*Thyroid hormone****s*:** Total T4 was determined through immunoassay, in which the control is combined with an enzyme-acceptor solution containing thyroxine antibody with a releasing agent, and an enzyme-donor solution containing enzyme substrate [4]. FTI was calculated from triiodothyronine (T3) uptake assay, in which ^125^I-labeled T3 is used as the tracer in the T3 uptake assay to fill the unbound thyroxine binding globulin (TBG) sites. T3 was determined by the Chiron Diagnostics ACS Chemiluminometric assay [5-7]. TSH was measured with a double antibody immunochemiluminescent procedure. The lower limit of sensitivity of this third-generation assay was 0.003 mcIU/ml.

**Analyses of Serum Hormones by Dr. Chatterton's Laboratory** :

*Sex hormone binding globulin (SHBG)*: The Delphia system was used for the assay of SHBG in serum. Materials were obtained from Wallac, Inc (Gaithersburg, MD). This is a solid phase two-site time-resolved fluoroimmunometric assay utilizing a sandwich technique. The limit of detection was 0.2 nmol/L and the intraassay and interassay coefficients of variation in assays [8] were 6% and 8% respectively.

*Dehydroepiandrosterone sulfate (DHEAS)*: DHEAS was measured in unextracted serum by radioimmunoassay as described previously [9]. Titrated tracers were obtained from Nuclear Corp, Boston, MA. Antiserum was obtained from ICN Biochemicals, Inc., Costa Mesa, CA. The limit of detection was 60 ng/ml and the intraassay and interassay coefficients of variation were 4.6% and 10.9%.

*Cortisol:* Plasma cortisol was measured by a direct assay described previously [10]. ^3^H-cortisol for the assay was obtained from New England Nuclear Div., Boston, MA. The limit of detection was 6 ng/ml and the intraassay and interassay coefficients of variation for cortisol in this assay were 8% and 11% respectively.

*Luteinizing Hormone (LH):* LH was measured by a coated tube radioimmunoassay. Materials were obtained from Diagnostics Products Corporation, Los Angeles. In this assay LH is captured between monoclonal anti-LH antibodies immobilized on the inside surface of the polystyrene tube and the ^125^labeled polyclonal anti-LH tracer. Results are expressed in mIU/ml in terms of the World Health Organization’s First International Reference preparation of LH for immunoassay, 68/40 (1^st^ IRP 68/40). The sensitivity is approximately 0.15 mIU/ml. Intra- and interassay coefficients of variation (CVs) average 3.0 and 7.1% respectively.

*Testosterone:* Testosterone was measured in serum by a coated tube assay obtained from Diagnostic Systems Laboratories, Webster, Texas. This assay employs ^125^I-testosterone as the tracer. The antiserum cross-reacts <0.9% with androstenedione and androstenediol, and 5.8% with dihydrotestosterone. Intra- and interassay coefficients of variation in previous assays were 4.9% and 7.5%.

*SHBG-Bound Testosterone:* SHBG-bound testosterone was determined as described by Bonfrer et al [11]. A 0.2 ml volume of serum diluted 1/8 with buffer is equilibrated with ^3^H-testosterone overnight at 4^0^C. A 0.10 ml suspension of a concavalin-A Sepharose conjugate (Pharmacia) is added to the serum. SHBG binds to the Con-A during a 30 min incubation period at room temperature. Testosterone in the serum maintains its equilibrium concentration with SHBG in the presence of endogenous factors such as other androgens, estrogens, and free fatty acids [12]. Separation of unbound ^3^H-testosterone from that bound to the Sepharose Con-A is achieved by centrifugation at 0^0^ C in order to minimize dissociation of bound estradiol. A pool of human serum is used as an internal control. The intra- and interassay CVs were 8.2 and 10.4% respectively.

*Estradiol*: Serum estradiol was measured using the Delphia procedure. Kits were obtained from Wallac, Inc., Gaithersburg, MD. The assay employed a sold-phase competitive immunoassay that employs a europium labeled estradiol and time-resolved fluorescence measurement for quantification. The limit of detection was 10 pg/ml. Intra- and interassay CVs for premenopausal women averaged 5.8% and 8.2% respectively.

*Insulin*: Plasma insulin was measured by an immunoradiometric assay (Diagnostic Products Corporation Los Angeles, CA) utilizing ^125^I-insulin. The sensitivity of the assay is 1.2 µIU/mL. All samples were measured in one assay; the intraassay CV was 5.1%.

*Quality control***:** External quality control of hormone assays standards were obtained from the American College of Pathologists. For internal quality control, a single batch of each of the quality control materials, antisera, and tracers for the assay of these analyses were prepared and reserved for all assays during the study. Intra-assay CVs were calculated from data in the study. The inter-assay CVs were calculated from data in the study and data from previously conducted assays to obtain sufficient numbers. Blood specimens were analyzed by technicians unaware of the participants' exposure group and 5% of the samples were submitted as blind duplicates to the laboratory as an additional quality control.

**Urine Analysis for 2-OHE_1_ and 16α-OHE_1_ by Dr. Bradlow's Laboratory**

Estrone metabolites, 2-hydroxyestrone and 16α – hydroxyestrone were measured directly and concurrently in urine with a commercially available EIA kit (Estramet 2/16, Immuna Care potation, Bethlemhem, PA) [13] previously described by Ziegler et al [14]. This assay uses specific murine monoclonal antibodies for each metabolite. They are bound to microliter plates. The enzyme alkaline phosphatase is linked to each metabolite, which competes with the metabolite:alkaline phosphatase to bind to the immobilized antibody. The rate of *p*-nitrophenol hydolysis is inversely related to the concentration of the metabolite in the sample. Estrogen metabolites were normalized using urine creatinine values (ng metabolite/mg creatinine). Urine creatinine was determined colorimetrically using a Beckman Creatinine II analyzer (Beckman Instruments, Brea California [15]. Limits of detection were 0.20 ng/ml. Within and between assay coefficients of variation for this assay are less than 9% [13].

References

1. Ballschmiter K, Zell M: **Analysis of polychlorinated biphenyls (PCB) by glass capillary gas chromatography.** *Fresenius J Anal Chem* 1980, **302:**20–31.
2. Burse VW, Groce DF, Korver MP, McClure PC, Head SL, Needham LL, Lapeza CR, Smrek AL: **Use of reference pools to compare the qualitative and quantitative determination of polychlorinated biphenyls by packed and capillary gas chromatography with electron capture detection. Part I.** *Serum Analyst* 1990, **115:**243-251.
3. Reimer CB, Smith SJ, Wells TW, Nakamura RM, Keitges PW, Ritchie RF, Williams GW, Hanson DJ, Dorsey DB: **Collaborative calibration of the U.S. National and the College of American pathologists reference preparations for specific serum proteins.** *Am J Clin Path* 1982, **77:** 12-19.
4. Henderson DR, Friedman SB, Harris JD, Manning WB, Zoccoli MA: **CEDIA, a new homogenous immunoassay system.** *Clin Chem* 1986, **32:**1637-1641.
5. Chodra IJ: **A radioimmunoassay for measurement of thyroxine in unextracted serum.** *J Clin Endocrinol Metab* 1972, **34:**938-947.
6. Hollander CS, Shenkman L, Mitsuma T, Asper SP: **Triodothyronine toxicosis developing during antithyroid drug therapy for hyperthyroidism.** *Johns Hopkins Med J* 1972, **132:**184-188.
7. Eastman CJ, Corcoran JM, Ekins RP, Williams ES, Nabarro JD: **The radioimmunoassay of triiodothyronine and its clinical application*.*** *J Clin Path* 1975, **28:**225-230.
8. Kowalski W, Chatterton R Jr: **Peripheral and not central suppression of ovarian function during osmotic pump infusion of ACTH (1-24) for one menstrual cycle in the cynomology monkey, and its partial compensation by a transitory elevation of sex hormone-binding globulin levels.** *Endocrinology* 1992, **130:**3582-3592.
9. Persky VW, Chatterton RT, Van Horn LV, Grant MD, Langenberg P, Marvin J: **Hormone levels in vegetarian and nonvegetarian teenage girls: potential implications for breast cancer risk.** *Cancer Res* 1992, **52:**578-583.
10. Chatterton RT Jr, Kazer RR, Rebar RW: **Depletion of luteal phase progesterone during constant infusion of cortisol phosphate in the cynomologus monkey.** *Fertil Steril* 1991, **56:**547-554.
11. Bonfrer JMG, Bruning PF, Nooijen WJ: **A simple method for the measurement of the steroid bound to sex hormone binding globulin in serum.** *J Steroid Biochem* 1989, **33**:227-231.
12. Street C, Howell RJS, Perry L, Al-Othman S, Chard T: **Inhibition of binding of gonadal steroids to serum binding proteins by non-esterified fatty acids: the influence of chain length and degree of unsaturation.** *Acta Endocrinol* 1989, **120:**175-179.
13. Klug TL, Bradlow TL, Sepkovic DW: **Monoclonal antibody-based enzyme immunoassay for simultaneous quantification of 2- and 16α-hydroxyestrone in urine.** *Steroids* 1994, **59:**648-655.
14. Ziegler RG, Rossi SC, Fears TR, Bradlow HL, Adlercreutz H, Sepkovic D, Kiuru P, Wahala K, Vaught JB, Donaldson JL, Falk RT, Fillmore CM, Siiteri PK, Hoover RN, and Gail MH: **Quantifying estrogen metabolism: An evaluation of the reproducibility and validity of enzyme immunoassays for the 2-hydoxy-estrone and the 16α hydroxyestrone in urine.** *Environ Health Perspect* 1997, **105:**607-614.
15. Flores OR, Sun L, Vaziri ND, Miyada DS: **Colorimetric rate method for the determination of creatinine as implemented by the Beckman Creatinine Analyzer 2.** *Am J Med Technol* 1980, **46:**792-798.

Table 1S

PCB Congener Limits of Detection (all values in ng/ml or ppb)

| **Analyte** | **Limit of Detection** |
| --- | --- |
| PCB028 | 0.03 |
| PCB052 | 0.02 |
| PCB056+060 | 0.08 |
| PCB066 | 0.05 |
| PCB074 | 0.05 |
| PCB099 | 0.05 |
| PCB101 | 0.05 |
| PCB105 | 0.05 |
| PCB110 | 0.06 |
| PCB118 | 0.05 |
| PCB130 | 0.12 |
| PCB137 | 0.10 |
| PCB138 | 0.03 |
| PCB146 | 0.04 |
| PCB149 | 0.08 |
| PCB153 | 0.04 |
| PCB156 | 0.03 |
| PCB157 | 0.07 |
| PCB167 | 0.10 |
| PCB170 | 0.03 |
| PCB171 | 0.08 |
| PCB172 | 0.06 |
| PCB177 | 0.05 |
| PCB178 | 0.05 |
| PCB180 | 0.02 |
| PCB183 | 0.05 |
| PCB187 | 0.03 |
| PCB189 | 0.04 |
| PCB191 | 0.08 |
| PCB193 | 0.06 |
| PCB194 | 0.04 |
| PCB195 | 0.04 |
| PCB201 | 0.06 |
| PCB203 | 0.04 |
| PCB205 | 0.09 |
| PCB206 | 0.02 |
| PCB208 | 0.08 |
| PCB209 | 0.06 |

Table 2S Relationships of Potential Confounders with Individual PCB Congeners, Pearson correlation coefficient, n=63

|  | 74^a^ | 99^a^ | 118^a^ | 138^a^ | 146^a^ | 153^a^ | 156^a^ | 170^a^ | 180^a^ | 187^a^ | 194^a^ | 201^a^ | 203^a^ | 206^a^ |
| --- | --- | --- | --- | --- | --- | --- | --- | --- | --- | --- | --- | --- | --- | --- |
| **Age** in years | .16 | **.27^b^** | **.32^b^** | **.36^b^** | **.41^b^** | **.36^b^** | **.43^b^** | **.34^b^** | **.30^b^** | **.33^b^** | **.36^b^** | **.31^b^** | **.28^b^** | **.27^b^** |
| BMI Group | -.12 | .08 | .06 | .03 | -.01 | .03 | -.07 | -.02 | .03 | .10 | -.06 | .04 | .11 | .04 |
| **Lipids** | .10 | .23 | .23 | .22 | **.27^b^** | .24 | .18 | **.26^b^** | **.26^b^** | **.30^b^** | .18 | **.25^b^** | **.28^b^** | .18 |
| **Drink Group** | -.23 | -.07 | -.18 | -.09 | -.17 | -.09 | -.18 | -.14 | -.11 | -.13 | -.18 | -.23 | -.17 | **-.27^b^** |
| GGT in U/L^a^ | .06 | .00 | -.02 | -.01 | .00 | -.02 | -.02 | -.01 | -.03 | -.04 | .00 | -.07 | -.09 | -.19^d^ |
| **TSH ultra sens** in mciu/mL^a^ | **-.34^bc^** | **-.25^bd^** | -.19 | **-.26^bd^** | **-.36^bc^** | -.24^d^ | **-.30^bc^** | **-.28^bd^** | -.20 | -.19 | -.21 | -.20 | -.14 | -.00 |
| Triiodothyronine in ng/dL^a^ | -.09 | -.04 | -.09 | -.02 | -.02 | -.01 | -.04 | .05 | .04 | .01 | -.00 | .07 | .09 | .07 |
| **T3-uptake** in **%** | **-.27^bc^** | -.21 | -.15 | -.20^d^ | -.18 | -.18 | -.17^d^ | **-.24^c^** | -.21^d^ | -.22^d^ | -.15 | -.22^d^ | **-.28^bc^** | -.18 |
| Total T4 in mcg/dL^a^ | .12 | .17 | .11 | .18 | .13 | .17 | .09 | .17 | .16 | .08 | -.01 | .04 | .12 | -.07 |
| Free T4 Index^a^ | -.02 | .05 | .03 | .06 | -.01 | .06 | -.01 | .01 | .01 | -.09 | -.16^d^ | -.16 | -.09 | -.17 |
| SHBG in nmol/L^a^ | -.01 | -.07 | -.02 | -.03 | .00 | -.03 | .08 | -.05 | -.10 | -.04 | .05 | .02 | -.08 | .08 |
| LH in mIU/ml^a^ | -.13 | -.16 | -.19^d^ | -.14 | -.10 | -.11 | -.10 | -.09 | -.11 | -.10 | -.06 | -.04 | -.04 | -.05 |
| **Testosterone (T)** in nmol/L^ae^ | -.06 | -.11 | .02 | -.01 | -.01^d^ | **.04^c^** | -.00 | **.07^c^** | **.08^c^** | -.05 | -.13 | -.06 | .02^d^ | -.04 |
| % T bound to SHBG | .04 | .08 | -.00 | .10 | .11 | .10 | .20 | .16 | .09 | .12 | .23 | .20 | .15 | .20 |
| **Conc. T** **bound to SHBG^a^** | -.05 | -.05 | .00 | .04 | .04 | .08 | .09 | **.13^c^** | **.11^c^** | .02 | .01 | .04 | .09^d^ | .05 |
| Estradiol (E2) in pmol/L | .16 | .09 | .19^d^ | .12 | .16^d^ | .15 | .04 | .13 | .15^d^ | .14 | .05 | .09 | .19^d^ | .04 |
| **DHEAS** in µmol/L^a^ | -.04 | .08 | .10^d^ | **.10^c^** | .05^d^ | **.12^c^** | -.02 | .10^d^ | **.17^c^** | .03 | -.07 | -.03 | .06 | -.08 |
| Cortisol in nmol/L^a^ | .13 | .12 | .16 | .14 | .16 | .14 | .13 | .11 | .16 | .15 | .17 | .17 | .17 | .09 |
| 2-OHE1 in ng/mL^a^ | .13 | .05 | -.02 | .03 | .02 | .00 | .03 | .01 | -.04 | -.10 | -.18 | -.18 | -.07 | -.15 |
| 16a-OHE1 in ng/mL^a^ | .04 | -.00 | .04 | .01 | -.01 | .00 | -.01 | .00 | -.03 | -.04 | -.06 | -.11 | -.00 | .03 |
| **2-OHE1/16a-OHE1**^a^ | .09 | .06 | -.10 | -.00 | .01 | -.02 | .01 | -.02 | -.04 | -.09 | -.16 | -.10 | -.13 | **-.28^bd^** |
| 2-OHE1/Creatinine^a^ | .03 | .03 | -.08 | -.00 | .05 | -.02 | .13 | .03 | -.03 | .03 | .10 | .09 | -.03 | .02 |
| 16a-OHE1/Creatinine^a^ | -.06 | -.02 | -.04 | -.03 | .03 | -.04 | .08 | .01 | -.04 | .09 | .19 | .16 | .04 | .22 |

^a^variable not normally distributed, therefore natural log-transformed variable used.

^b^p-value<.05 in univariate analysis.

^c^p-value<.05 after control for age, bmi group and total lipids.

^d^p-value≥.05, but less than .10 after control for age, bmi group and total lipids.

^e^Significant associations between testosterone and congeners were positively (and not negatively) related.
